# Bleomycin promotes cellular senescence and activation of the cGAS-STING pathway without direct effect on fibrosis in an idiopathic pulmonary fibrosis model

**DOI:** 10.18632/aging.206312

**Published:** 2025-08-28

**Authors:** Yoko Miura, Nadia Milad, Akiho Takata, Satoshi Kanazawa

**Affiliations:** 1 Department of Neurodevelopmental Disorder Genetics, Nagoya City University Graduate School of Medical Sciences, Nagoya, Japan; 2 Department of Medicine, McMaster University, Hamilton, Canada; 3 Department of Medicine, Division of Respirology, Firestone Institute for Respiratory Health, McMaster University, Hamilton, Canada

**Keywords:** bleomycin, cGAS-STING, idiopathic pulmonary fibrosis (IPF), precision-cut lung slices (PCLS), senescence

## Abstract

Bleomycin is an effective anticancer agent that causes drug-induced interstitial pneumonia (IP). Medical history is a risk factor for adverse effects, particularly a history of IP and age-related fibrosis. Anti-cancer drugs for lung cancer with idiopathic pulmonary fibrosis (IPF) often aggravate pulmonary fibrosis. Thus, we examined the pathological effects of bleomycin, an anticancer drug, in precision-cut lung slices (PCLS) of lungs with usual interstitial pneumonia (UIP). We found that the lungs of mice with induced UIP (iUIP), which exhibit a pathology similar to that of IPF, underwent accelerated senescence. Treatment of iUIP PCLS with bleomycin reduced the nuclear membrane component lamin B1 and nuclear DNA with γH2AX leaked into the cytoplasm. This perinuclear DNA may activate NF-κB through the cyclic GMP-AMP synthase-stimulator of interferon genes (cGAS-STING) pathway. As a result, the unresolved DNA damage associated with the failure of DNA repair and senescence progression is more advanced in these cells. However, *Col1a1* and *Acta2* expression was not induced in either bleomycin-treated normal or iUIP PCLS, suggesting that there was no direct fibrotic effect on the lungs. We concluded that lungs with iUIP exhibited accelerated senescence following bleomycin treatment, leading to cell death.

## INTRODUCTION

Bleomycin, a glycopeptide antibiotic produced by *Streptomyces verticillus*, serves as a therapeutic agent for malignant tumors and is a causative agent of interstitial pneumonia (IP) development [1]. Patients treated with bleomycin as a neoplastic therapy often develop progressive pulmonary fibrosis, which is consistent with animal models [2, 3]. This is due to bleomycin’s ability to induce cell injury, most likely necrosis, through double-stranded DNA breaks (DSBs) [4, 5]. However, the precise reason patients with idiopathic pulmonary fibrosis (IPF) exhibit heightened sensitivity to drug-induced IP remains unclear. The mechanism underlying bleomycin-induced DSBs involves the formation of a complex between bleomycin and Fe^2+^ via the generation of reactive oxygen species (ROS), resulting in a DNA damage response (DDR) and ER stress [6–8]. This cascade activates the protein kinase ataxia telangiectasia to be mutated in response to DSBs, triggering the phosphorylation of histones H2AX, Chk2, p53, and MDM2 to facilitate DNA repair. Notably, the phosphorylation of serine 139 on histone H2AX, known as γH2AX, serves as a marker for DSBs and has been detected after day 3 in *in vivo* studies involving bleomycin treatment [9–11]. In addition, bleomycin has been shown to transiently induce *Col1a1* in *in vitro* studies using fibroblasts [12, 13]. However, the direct effect of bleomycin on fibroblasts was transient, ultimately reducing *Col1a1* expression within 24 hours. *In vivo* studies have shown that intratracheal instillation of bleomycin affects type I and II alveolar epithelial cells (AEC1 and AECII) but has no direct effect on interstitial pulmonary fibroblasts [9, 14]. In these studies, histological characteristics of the lungs showed minimal inflammation and fibrosis symptoms. Therefore, the effect of bleomycin on the entire lung in the acute, primary phase within one week after bleomycin administration remains unclear.

IPF is a fatal disease characterized by chronic and progressive fibrosis [15]. Histologically, it is characterized by usual interstitial pneumonia (UIP) and severe fibrosis with a honeycomb structure. Lung fibrosis is associated with aging because telomere shortening in alveolar epithelial cells promotes lung remodeling [16]. It has been suggested that cellular senescence causes pulmonary dysfunction [14]. Various senescence markers and senescence-associated secretory phenotype (SASP) factors, such as matrix metalloproteinases (MMPs) have been detected in alveolar epithelial cells and fibroblasts. The cytoplasmic DNA sensing molecule cyclic GMP-AMP synthase (cGAS) detects cytosolic chromatin fragments in senescent cells, leading to the activation of the cGAS stimulator of the interferon gene (STING) pathway. A decrease in lamin B1, a component of the nuclear membrane, is a hallmark of cellular senescence [17]. This results in self-chromatin leakage into the cytoplasm and ultimately promotes the release of SASP factors from senescent cells through the NF-κB cascade of the cGAS-STING pathway [18–20]. STING triggers an innate immune response; thus, self-DNA sensing during aging is involved in inflammation and fibrosis [21, 22].

In this study, we used lung tissue culture *ex vivo* models, known as precision-cut lung slices (PCLS), and lung sections. Originally developed to analyze bronchoconstriction-induced effects, this technique has been used to evaluate chemical toxicity [23]. Recent studies using PCLS have used conventional bleomycin-induced fibrosis animal models and human specimens to assess fibrosis and the effects of therapeutic agents such as bleomycin [24]. PCLS consists of thin slices of lung tissue that preserve the structural integrity of the lungs, making it suitable for the assessment of lung damage. To better understand the mechanism of drug-induced lung injury associated with IP, we used the *ex vivo* PCLS culture system of our induced-usual interstitial pneumonia (iUIP) mouse model [9]. We found that in lungs with iUIP, where cellular senescence has already been initiated, bleomycin treatment induces nuclear membrane fragility, thereby impeding DNA repair mechanisms. Interestingly, increased cellular senescence promotes acute exacerbation by augmenting cell death, suggesting directions for developing therapeutic strategies for the treatment of drug-induced IP. Furthermore, as we saw only transient increases in collagen expression, our data suggest that bleomycin does not directly induce fibrosis.

## RESULTS

### Lungs with iUIP undergo accelerated cellular senescence

**Figure 1:**
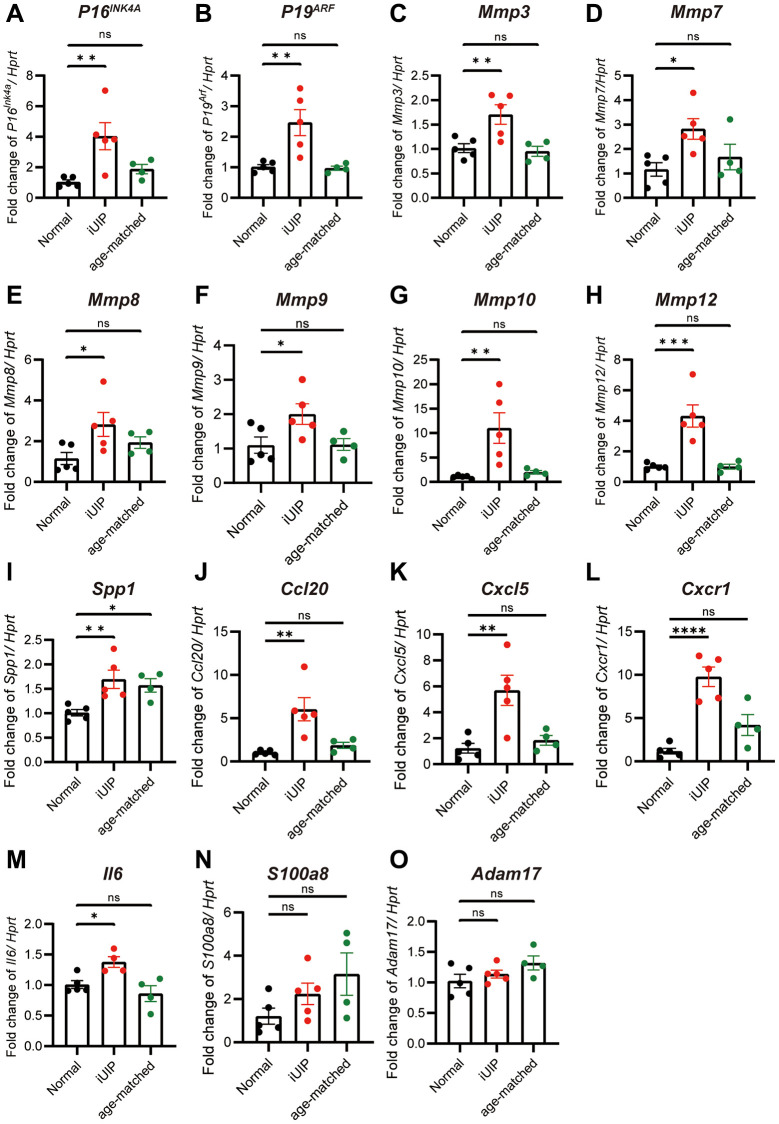
**Lungs with iUIP were already in senescence state.** Total RNA was extracted from iUIP, normal mice (healthy control mice with the same genetic background as iUIP mice), and age-matched control lungs. *P16^INK4A^* (**A**), *P19^ARF^* (**B**), *Mmp3* (**C**), *Mmp7* (**D**), *Mmp8* (**E**), *Mmp9* (**F**), *Mmp10* (**G**), *Mmp12* (**H**), *Spp1* (**I**), *Ccl20* (**J**), *Cxcl5* (**K**), *Cxcr1* (**L**), *Il6* (**M**), *S100a8* (**N**), and *Adam17* (**O**) expression as determined by qPCR. *Hprt* served as an internal control. The results are shown as mean ± SE of five mice at normal and iUIP and four mice at age-matched control. Asterisks indicate ^*^P < 0.05, ^**^P < 0.01, ^***^P < 0.001, and ^****^P < 0.0001 compared with normal lungs. “ns”, not statistically significant.

As the lungs of patients with IPF are characterized by their accelerated senescence [16], we examined whether the lungs in the iUIP mouse model were also in a state of cellular senescence. We examined the expression of two key cellular senescence markers, *P16^INK4A^* and *P19^ARF^*. When comparing lungs with iUIP to healthy lungs, both *P16^INK4A^* and *P19^ARF^* mRNA expression levels were significantly increased (Figure 1A, 1B). Furthermore, mRNA expression of various *Mmps* including *Mmp3*, *Mmp7*, *Mmp8*, *Mmp9*, *Mmp10*, *Mmp12* as well as related SASPs, including *Spp1, Ccl20*, *Cxcl5*, and *Cxcr1* and interleukin-6 (*Il6)* were increased in iUIP lungs (Figure 1C–1M) [25]. The expression of *S100a8* was increased, but comparable to age-matched control (Figure 1N). Both *Cxcl5* and *Ccl20* are primarily expressed in type II alveolar epithelial cell, while *S100a8* is predominantly expressed in neutrophils and macrophages. Because Adam17 produces soluble IL6r, its mRNA levels were not elevated (Figure 1O). Interestingly, the increased expression of senescence markers and SASP factors in lungs with iUIP was not observed in age-matched control lungs. This suggests that senescence is already specifically occurring in lungs with iUIP and is not merely a consequence of aging or genetic background.

### Bleomycin did not directly induce fibrosis in either bleomycin-treated normal or iUIP PCLS

**Figure 2:**
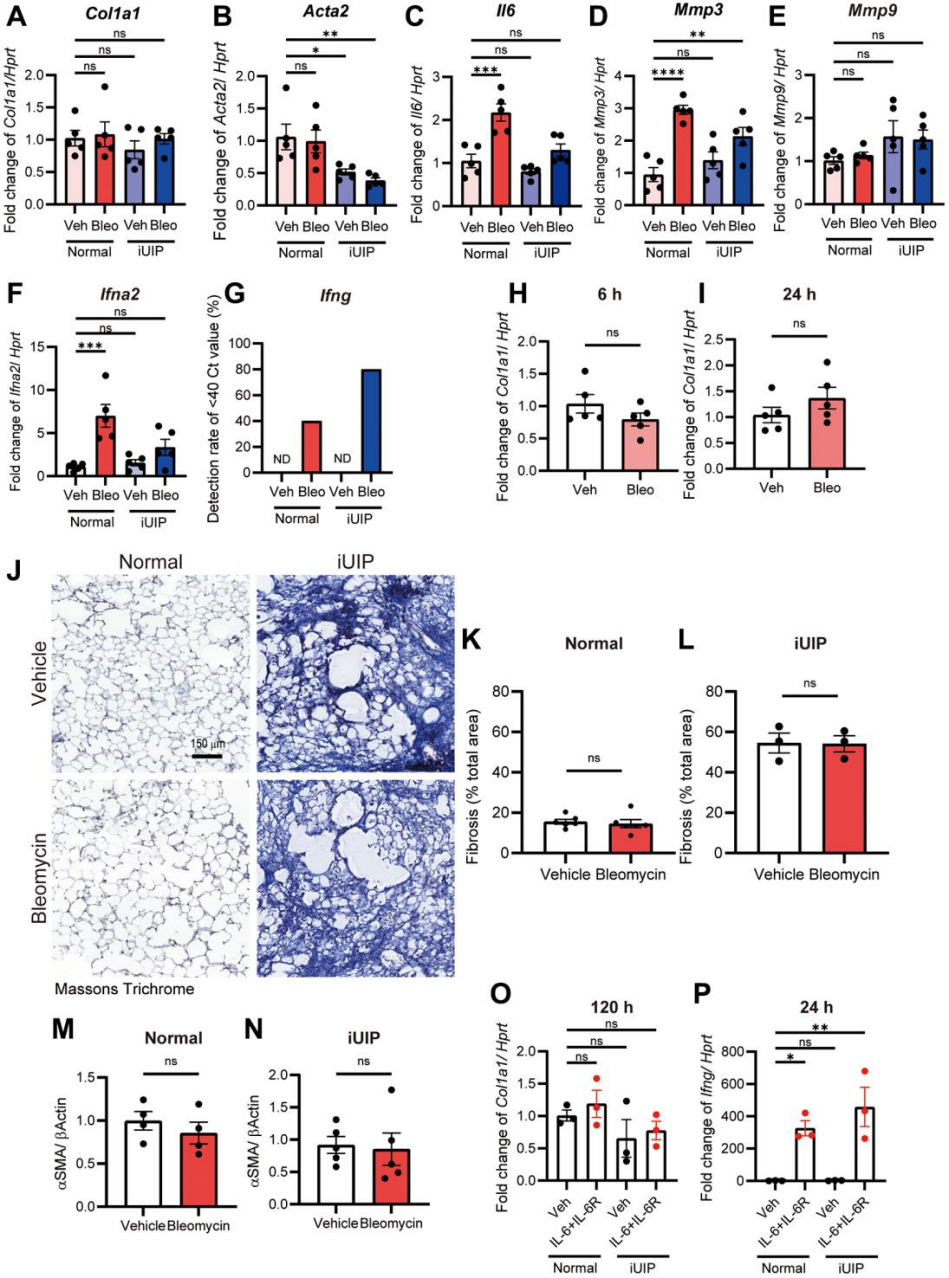
**Bleomycin treatment did not directly induce fibrosis in normal and iUIP PCLS.** (**A**–**G**) Normal and iUIP PCLS samples were treated with the vehicle or 1 μM bleomycin for 120 h. The expression levels of *Col1a1* (**A**), *Acta2* (**B**), *Il6* (**C**), *Mmp3* (**D**), *Mmp9* (**E**), *Ifna2* (**F**), and *Ifng* (**G**) were determined using qPCR. *Ifng* expression was not detected in vehicle-treated normal and iUIP PCLS. The detection rates of samples with Ct values <40 are shown in graph (**G**). (**H**, **I**) PCLS samples were treated with the vehicle or 1 μM bleomycin for 6 (**H**) and 24 h (**I**). The expression of *Col1a1* was determined using qPCR. (**J**) Masson’s trichrome staining of PCLS from normal and iUIP treated with the vehicle or 1 μM bleomycin for 48 h. (**K**–**N**) Percentage of fibrosis area ratio (**K**, **L**) were calculated based on the total area in each PCLS. (**M**, **N**) aSMA expression was determined using WB in PCLS from normal and iUIP treated with the vehicle or 1 μM bleomycin for 48 h. β-Actin was used as an internal control for WB. (**O**, **P**) PCLS samples were treated with vehicle or IL-6 (50 ng/mL) and IL-6R (50 ng/mL) for 120 (**O**) and 24 h (**P**). The expression of *Col1a1* (**O**) and *Ifng* (**P**) was determined using qPCR. *Hprt* served as an internal control. The results are shown as mean ± SE of *n* = 5 mouse PCLSs at each stage (*n* = 3 mouse PCLS for iUIP fibrosis and *n* = 4 of normal in aSMA WB (**M**)). Asterisks indicate ^*^P < 0.05, ^**^P < 0.01, ^***^P < 0.001, and ^****^P < 0.0001 compared with vehicle-treated normal PCLS. “ns”, not statistically significant. Scale bar indicates 150 μm.

Some anticancer drugs cause exacerbations, including lung fibrosis, which impair gas exchange and cause respiratory failure. To determine whether bleomycin directly caused fibrosis, we examined collagen type I (*Col1a1*) and alpha smooth muscle actin (*Acta2*) mRNA expression levels in bleomycin-treated PCLS. After 120 h, the mRNA levels of *Col1a1* and *Acta2* remained unchanged in both normal and iUIP PCLS (Figure 2A, 2B). In contrast, *Il6*, *Mmp3*, and *Ifna2* expression levels were increased in bleomycin-treated normal and iUIP PCLS, except for *Mmp9* (Figure 2C–2F). Interestingly, *Ifng* expression was below the detection limit of qPCR in both normal and iUIP PCLS at 120 h. However, bleomycin treatment induced *Ifng* expression to levels detectable by qPCR (Figure 2G). Since bleomycin treatment transiently increases *Col1a1* mRNA expression in human dermal fibroblasts [12], we examined whether the fibrotic response to bleomycin treatment was more rapid in PCLS. However, no changes in *Col1a1* expression were observed after 6 h or 24 h of PCLS culture with bleomycin (Figure 2H, 2I). Quantification of Masson’s trichrome staining in histopathology showed that, although percentage fibrosis area in the lung was substantially higher in iUIP lungs, treatment of PCLS with bleomycin had no effect on collagen deposition in normal or iUIP slices (Figure 2J–2L). Similarly, Col1a1 and alpha smooth muscle actin (αSMA) staining by immunohistopathology was similar between vehicle- and bleomycin-treated PCLS (Supplementary Figure 1). Western blot (WB) data for αSMA also showed the same result (Figure 2M, 2N, and Supplementary Figure 2A). Since frozen-thawed PCLS was used in the experiments, we assessed tissue viability and integrity, finding no significant changes in cell death, in lung structure or in cell populations such as epithelial cells, fibroblasts, or lymphocytes (Supplementary Figure 3). Thus, in the PCLS model, which reflects the whole lung rather than single cultured cells, bleomycin induced inflammation, including increased *Il6* expression, without directly inducing fibrosis and collagen deposition.

Previous work has shown that iUIP PCLS respond to a fibrosis cocktail containing transforming growth factor-β (TGF-β), where lungs in this UIP phase were found to be more susceptible to fibrosis [26, 27]. Therefore, we examined the combination of IL-6 and IL-6R as another fibrotic effector in PCLS. However, *Col1a1* expression was not induced at 24 h, 48 h, or 120 h (Figure 2O and Supplementary Figure 4A, 4B) [28, 29]. Furthermore, the expression of *Ifng*, a putative anti-fibrotic effector, was increased by the combination of IL-6 and IL-6R in normal and iUIP PCLS (Figure 2P). Thus, these results suggest that the lung is intrinsically resistant to fibrosis owing to the lack of acute fibrotic response to bleomycin.

### Bleomycin treatment generated ROS in both normal and iUIP PCLS but only iUIP PCLS were susceptible to cytotoxicity

**Figure 3:**
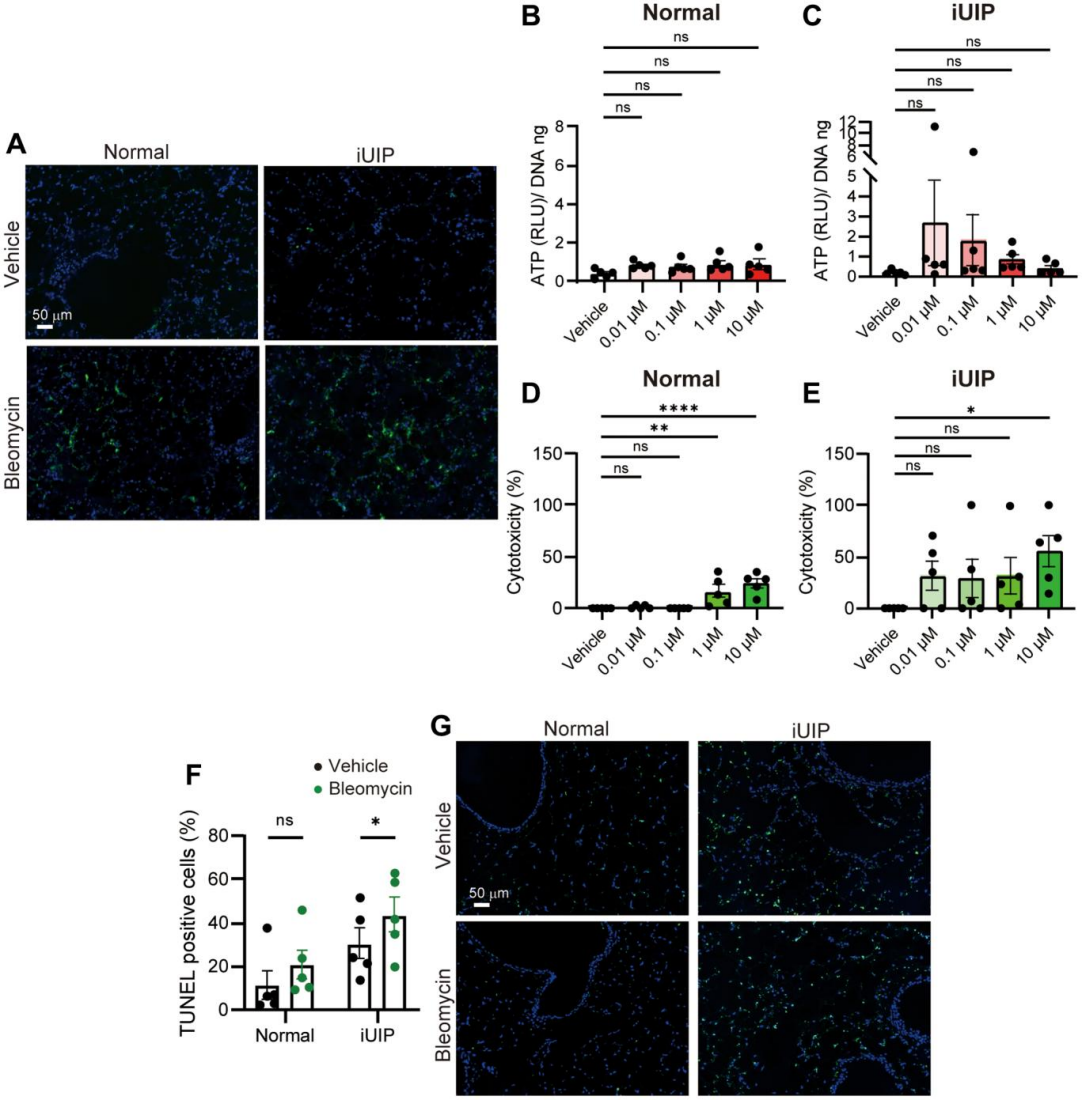
**Cellular cytotoxicity was induced by bleomycin treatment in PCLS from iUIP mice.** (**A**) ROS generation was detected in PCLS from normal and iUIP mice treated with the vehicle or 1 μΜ bleomycin for 30 min. (**B**, **C**) Extracellular ATP was examined in PCLS from normal (**B**) and iUIP (**C**) treated with the vehicle or 0.01–10 μΜ bleomycin for 4 h. Values were normalized to DNA content. (**D**, **E**) LDH assay was examined in PCLS from normal (**D**) and iUIP (**E**) treated with the vehicle or 0.01–10 μM bleomycin for 48 h. Values were normalized to DNA content. (**F**, **G**) PCLS samples were treated with 1(μM bleomycin for 120 h. TUNEL-positive cells in paraffin-embedded sections were stained and calculated based on the total number of cells. The results are shown as mean ± SE of five mouse PCLSs at each stage. Asterisks indicate ^*^P < 0.05, ^**^P < 0.01, and ^****^P < 0.0001 compared with the vehicle. “ns”, not statistically significant. Scale bars indicate 50 μm.

Bleomycin induces ROS generation, resulting in DSBs as the first rapid reaction. To evaluate ROS generation, *ex vivo* cultures of iUIP and normal PCLS were performed. Histopathological analysis revealed abundant ROS generation with bleomycin treatment, but no difference between normal and iUIP PCLS treated with 1 μΜ bleomycin for 30 min was observed (Figure 3A).

Extracellular ATP, a “find-me” signal of early cell death, was measured in PCLS incubated with various bleomycin concentrations for 4 h. Extracellular ATP was detected at low levels in iUIP and normal PCLS, as well as in age-matched controls, even with very low concentrations (0.01 μM) of bleomycin (Figure 3B, 3C and Supplementary Figure 5A). Baseline extracellular ATP, which reflects cellular activity, was lower in iUIP PCLS than in normal and age-matched controls (Supplementary Figure 5B).

Extracellular LDH levels were also measured as a molecular event of late cell death. iUIP PCLS were damaged by low bleomycin concentrations, making BLM-induced cell death more evident (Figure 3D, 3E). In age-matched controls, the extracellular LDH levels were similar to those in normal lungs (Supplementary Figure 5C). We conclude that iUIP PCLS are more sensitive to bleomycin cytotoxicity than normal and age-matched control PCLS.

Further examination using the TdT-mediated dUTP nick labeling (TUNEL) assay revealed that bleomycin-induced cell death was more prevalent in the alveolar cells of iUIP PCLS than in those of normal PCLS. Bleomycin affected the death of alveolar cells in the lumen, particularly in iUIP PCLS, but not in bronchiolar epithelial cells at 120 h (Figure 3F, 3G). These positive cells were observed after 120 h, but not after 48 h of incubation (Figure 3G and Supplementary Figure 6). Collectively, these data indicate that bleomycin treatment causes similar levels of ROS generation in both normal and iUIP PCLS; however, iUIP PCLS are highly susceptible to cytotoxicity.

### Bleomycin reduced cell viability in iUIP PCLS

**Figure 4:**
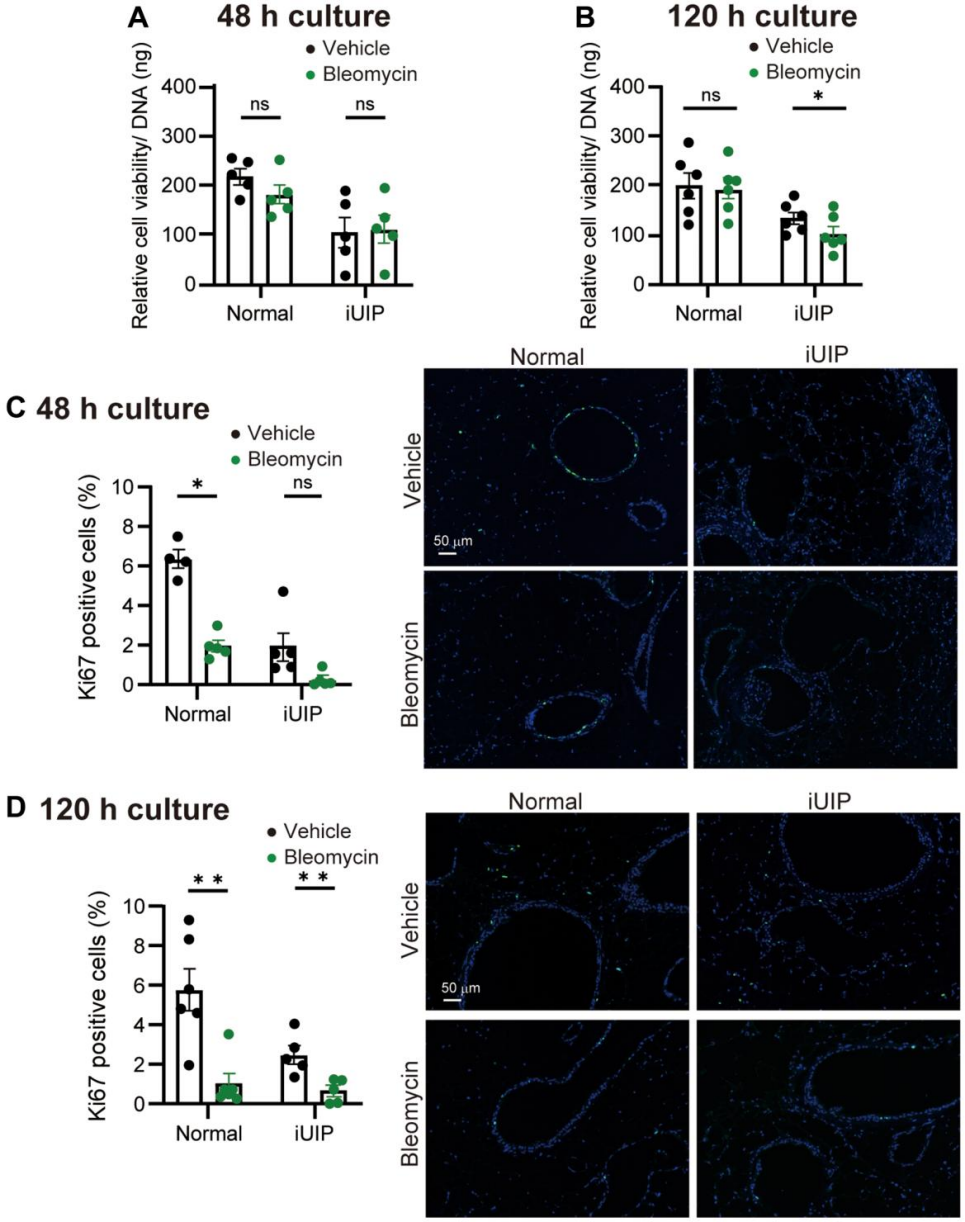
**Viability was reduced after bleomycin treatment in PCLS from iUIP mice.** (**A**, **B**) Relative viability was examined in PCLS from normal and iUIP treated with the vehicle or 1 μΜ bleomycin for 48 (**A**) or 120 h (**B**). Values were normalized to DNA content. (**C**, **D**) Ki67-positive cells in paraffin-embedded sections were stained and calculated based on the total number of cells. The results are shown as mean ± SE of five mouse PCLSs at each stage. Asterisks indicate ^*^P < 0.05 and ^**^P < 0.01 compared with the vehicle. “ns” is not statistically significant. Scale bars indicate 50 μm.

Next, normal and iUIP PCLS were cultured for 48 and 120 h to compare the effects of bleomycin on cell viability and cell division. Relative cell viability was reduced only in iUIP PCLS after 120 h but was unchanged at 48 h, without significant effect in control PCLS at any timepoint (Figure 4A, 4B). In addition, we examined the percentage of Ki67-positive cells at 48 and 120 h in normal and iUIP PCLS to assess cell proliferation. The number of Ki67 positive cells at baseline in iUIP PCLS was less than half of that in normal PCLS group (Figure 4C, 4D). Nevertheless, the percentage of Ki67 positive cells was reduced in both normal and iUIP PCLS (trend) following bleomycin treatment, with iUIP PCLS proliferation almost entirely abolished. Thus, it seems that iUIP PCLS were more susceptible to bleomycin treatment-induced cell death and impaired cell proliferation after 48 h.

### Bleomycin treatment altered the localization of DNA damage repair marker γH2AX in iUIP PCLS

**Figure 5:**
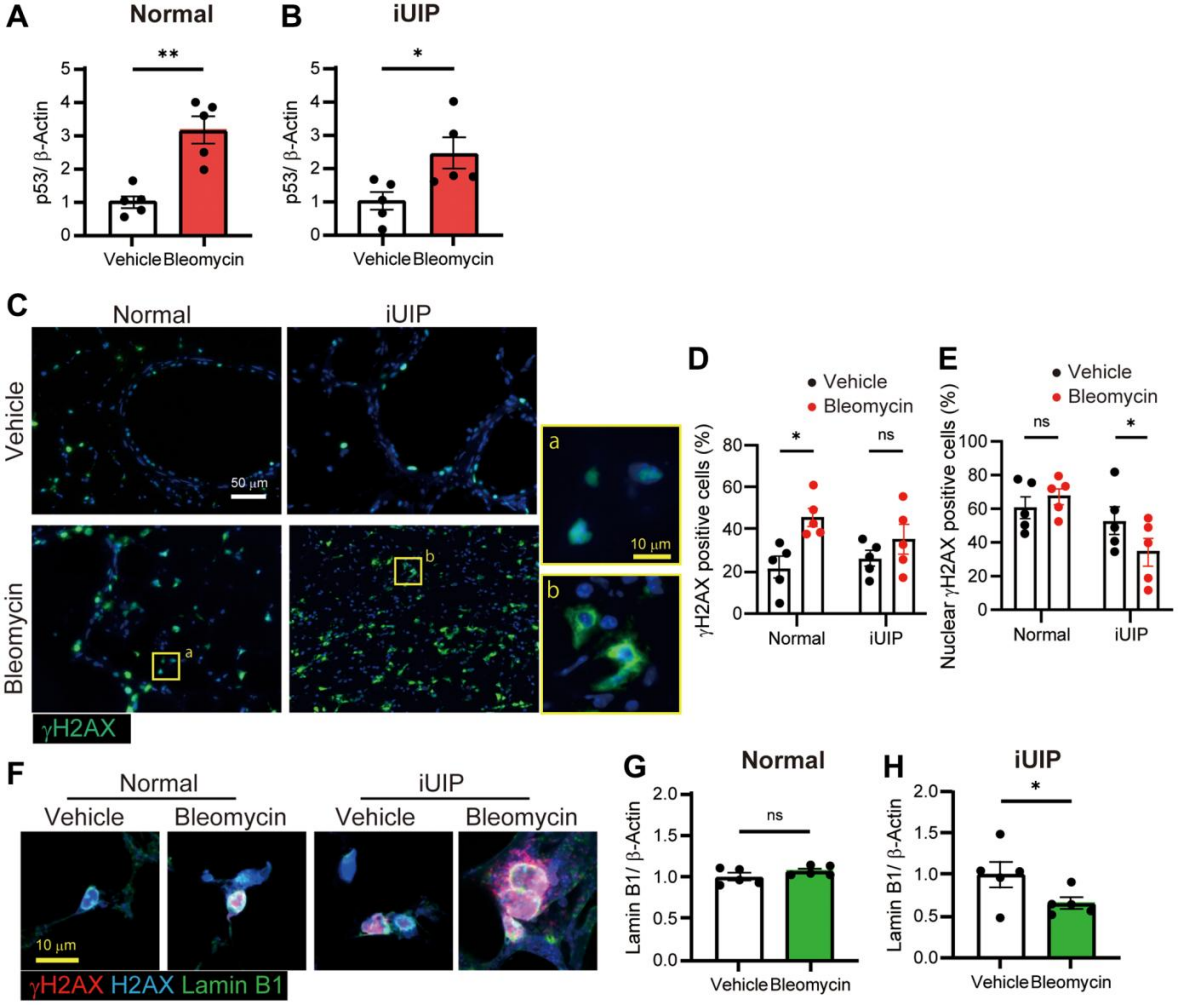
**γH2AX expression was observed in the perinuclear region of the iUIP PCLS.** (**A**, **B**) p53 expression was determined using WB in PCLS from normal (**A**) and iUIP (**B**) treated with the vehicle or 1 μΜ bleomycin for 48 h. (**C**) IF staining for γH2AX in PCLS paraffin-embedded sections from normal and iUIP treated with the vehicle or 1 μM bleomycin for 48 h. (**D**, **E**) Percentage of γH2AX-positive cells (**D**) and nuclear γH2AX-positive cells (**E**) were calculated based on the total number of cells. (**F**) IHC staining for(γH2AX (red), H2AX (blue), and lamin B1 (green) in PCLS from normal and iUIP treated with the vehicle or 1 μM bleomycin for 48 h. (**G**, **H**) Lamin B1 expression was determined using WB in PCLS from normal (**G**) and iUIP (**H**) treated with the vehicle or 1 μM bleomycin for 48 h. β-Actin was used as an internal control for WB. The results are shown as mean ± SE of five mouse PCLSs in each group. Asterisks indicate ^*^P < 0.05 and ^**^P < 0.01 compared with the vehicle. “ns”, not statistically significant. Scale bars indicate 50 μm (white) and 10 μm (yellow). Immunohistochemical staining of gH2AX was performed three times independently.

To evaluate the DNA-damage response, we examined the expression of p53 in bleomycin-treated PCLS. The expression of p53 was increased in both normal and iUIP PCLS cells (Figure 5A, 5B and Supplementary Figure 2B). The phosphorylation of Ser139 of histone H2AX to form γH2AX is a critical step in DNA damage repair, and bleomycin treatment induces the accumulation of nuclear γH2AX when administered *in vivo* [9]. Most γH2AX-positive cells were observed in bronchiolar epithelial cells and alveoli of the lungs in both PCLS (Figure 5C, 5D). In contrast, the cellular localization of γH2AX differed in iUIP PCLS, with most γH2AX localized diffusely in the perinuclear region, suggesting disruption of nuclear membrane integrity. Bleomycin-treatment of iUIP PCLS led to reduced numbers of γH2AX-positive cells in the nucleus (Figure 5E).

DSBs alter the profiles of senescence-related events, such as decreasing lamin B1 expression. To confirm that γH2AX localization in the perinuclear region may occur due to disrupted nuclear membrane function, we performed immunohistochemical (IHC) staining for lamin B1, γH2AX, and histone H2AX in bleomycin-treated PCLS from iUIP and controls. Both γH2AX and histone H2AX were extended to the perinuclear region outside the lamin B1-positive nuclear membrane (Figure 5F). In bleomycin-treated iUIP PCLS, the nuclei were expanded and the expression of lamin B1 was not fully circular. The significant decrease in lamin B1 expression, a cellular senescence criterion, was confirmed using WB in bleomycin-treated iUIP PCLS (Figure 5G, 5H). No such reduction in lamin B1 expression was observed in lungs with iUIP (Supplementary Figures 7 and 2C). Collectively, these results indicate that damage responses and senescence were exacerbated by bleomycin treatment in iUIP PCLS.

### Perinuclear DNA activates cGAS-STING pathway

**Figure 6:**
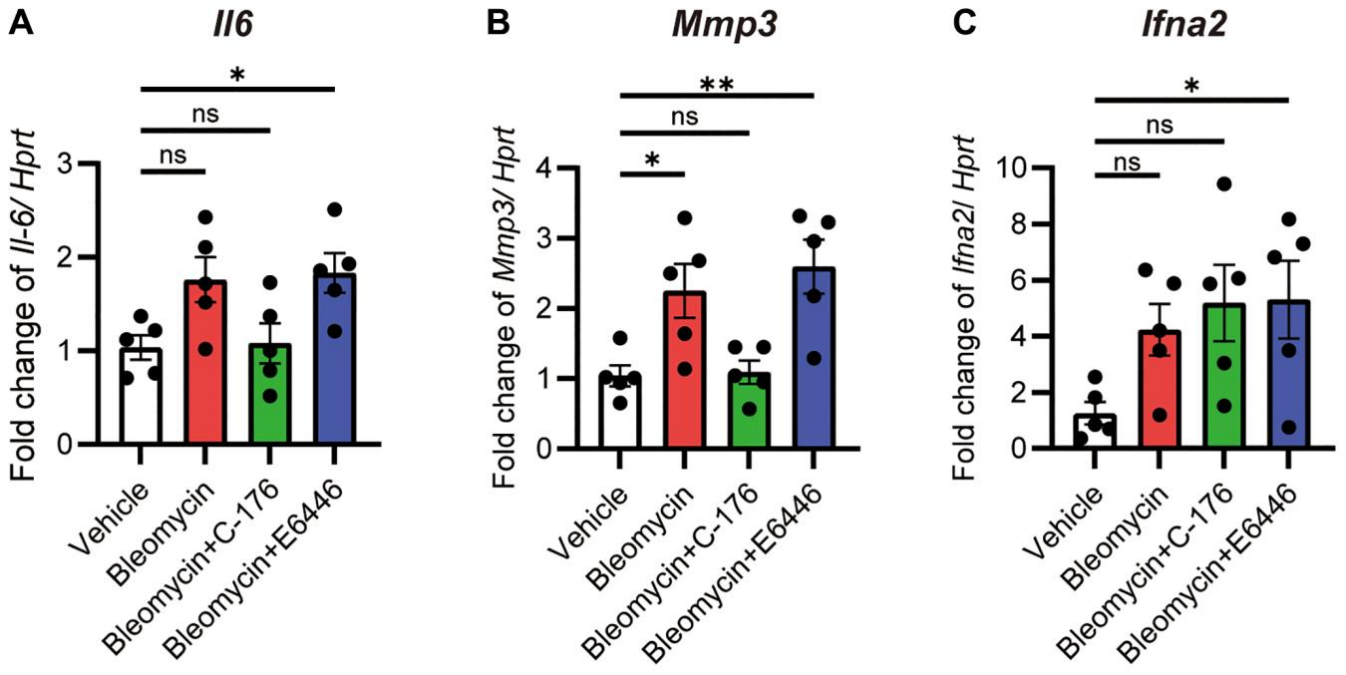
**The cGAS-STING pathway was activated after bleomycin treatment.** PCLS samples from the iUIP were treated with 1 μM bleomycin mixed with 1 μM C-176 or E6446 dihydrochloride for 120 h. *Il6* (**A**), *Mmp3* (**B**), and *Ifna2* (**C**) mRNA expression levels were determined using qPCR. *Hprt* served as an internal control. The results are shown as mean ± SE of five mouse PCLSs at each group. Asterisks indicate ^*^P < 0.05, ^**^P < 0.01, compared to the vehicle. “ns”, not statistically significant.

The cGAS-STING pathway recognizes DNA fragments in the cytoplasm and induces the expression of NF-κB and interferon regulatory factor-3 (IRF3) target genes, including *Il6*, *Mmp* genes, and type I interferon (*Ifnα2*). As *Il6* and *Mmp3* were increased in bleomycin-treated iUIP PCLS (Figure 2C, 2D), the expression of *Il6* and *Mmp3* was inhibited by C-176 treatment (STING pathway blocker) but not by E6446 dihydrochloride (TLR pathway blocker) (Figure 6A, 6B). In contrast, *Ifna2* was slightly upregulated by bleomycin treatment, an effect that was not completely blocked by either inhibitor (Figure 6C). Therefore, it seems that the leakage of DNA fragments into the cytoplasm caused by bleomycin treatment was the result of decreased lamin B1 and subsequent activation of the cGAS-STING pathway.

### Cellular senescence was exacerbated in iUIP PCLS treated with bleomycin

**Figure 7:**
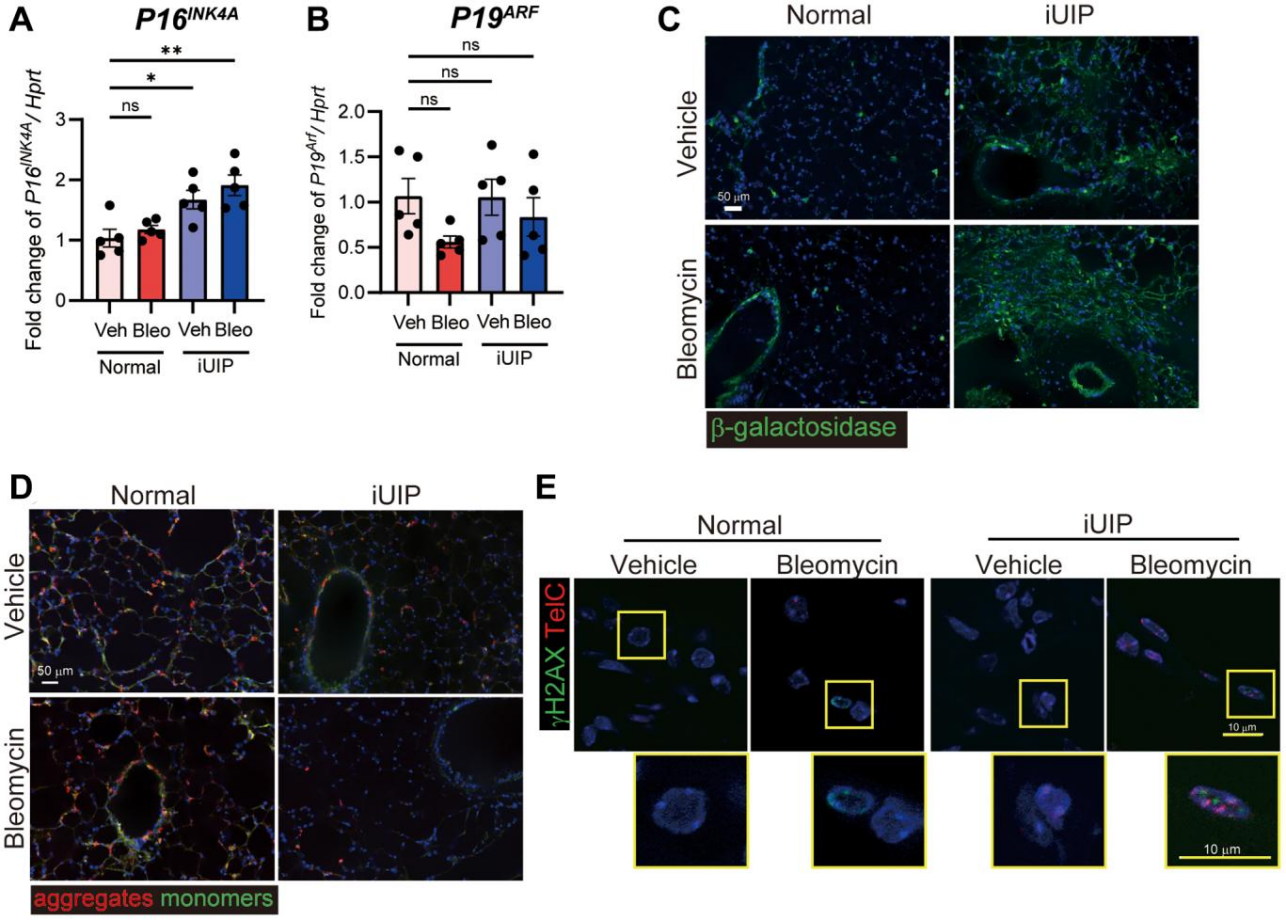
**Excessive senescence occurred in iUIP PCLS treated with bleomycin.** (**A**, **B**) PCLS samples were treated with the vehicle or 1 μM bleomycin for 120 h. *P16^INK4A^* (**A**) and *P19^ARF^* (**B**) expression were determined using qPCR. *Hprt* was used as an internal control for qPCR. The results are shown as mean ± SE of five mouse PCLSs at each stage. Asterisks indicate ^*^P < 0.05 and ^**^P < 0.01 compared with the vehicle. “ns”, not statistically significant. (**C**) β-galactosidase staining was detected in PCLS from normal and iUIP was treated with the vehicle or 1 μΜ bleomycin for 48 h. (**D**) JC-1 staining was detected in PCLS from normal and iUIP was treated with the vehicle or 1 μΜ bleomycin for 4 h. (**E**) TAFs (TelC, red) were co-localized with γH2AX (green) using IF and telomere *in situ* hybridization. Scale bars indicate 50 μm (white) and 10 μm (yellow).

In iUIP PCLS, bleomycin decreases lamin B1 and extends perinuclear γH2AX expression. As this phenomenon indicates enhanced cellular senescence in iUIP PCLS, we confirmed *P16^INK4A^* and *P19^ARF^* expressions following bleomycin treatment in normal and iUIP PCLS. The *P16^INK4A^* expression was increased in iUIP PCLS compared to normal PCLS and was further enhanced by bleomycin treatment in iUIP but not in normal PCLS (Figure 7A). The *P19^ARF^* expression was not increased by bleomycin treatment (Figure 7B). Histological analysis showed that the senescence biomarker, β-galactosidase staining was stronger in iUIP PCLS than in normal PCLS (Figure 7C). Upon closer inspection, positive staining was observed primarily in bronchiolar epithelial cells and macrophages. It was also observed in alveolar regions consisting mainly of type I and type II epithelial cells in both PCLS. These data indicate that senescence through *P16^INK4A^* was accelerated in iUIP PCLS treated with bleomycin.

Next, we conducted JC-1 staining to assess mitochondrial membrane potential in PCLS. JC-1 staining was lower in vehicle-treated iUIP PCLSs than in normal and age-matched control samples. Bleomycin treatment exacerbated JC-1 staining, indicating a potential reduction in the cellular activity of iUIP PCLS (Figure 7D and Supplementary Figure 8). Next, we examined telomere-associated foci (TAF) using TelC, a senescence marker that identifies unresolved DNA damage sites within telomeres. In bleomycin-treated iUIP PCLS, TAFs were co-localized with γH2AX by immunofluorescence (IF) and telomere *in situ* hybridization, unlike in normal PCLS treated with bleomycin (Figure 7E). Therefore, these data indicate that lungs with iUIP undergoing cellular senescence exhibit increased senescence after bleomycin treatment.

## DISCUSSION

We previously established an iUIP mouse model by combining the conventional bleomycin method with a mouse model of rheumatoid arthritis associated with lung disease. First, mice were intratracheally administered bleomycin (approximately 1/3 of the conventional dose) to induce UIP-like pathological features in the lungs. These lungs with iUIP already exhibited signs of cellular senescence, similar to those of patients with IPF [9]. In addition, lung cancer as a complication of IPF is treated with anticancer drugs that can sometimes induce fatal exacerbations. In this study, to clarify the pathophysiological features of drug-induced exacerbation of IPF in the lungs, we used *ex vivo* cultures of PCLS from iUIP mice as well as control mice and examined the effects of bleomycin treatment *ex vivo* in the IPF model. Bleomycin induces DSB by producing ROS, where we observed similar ROS generation in normal and iUIP PCLS. However, TUNEL positive cells increased only in iUIP PCLS. These results indicate that the failure to repair DNA damage induced by bleomycin led to increased cell death primarily in iUIP PCLS. Histological and biochemical analyses demonstrated that the nuclear membrane component lamin B1 was diminished by bleomycin treatment, and nuclear DNA containing γH2AX was released into the perinuclear space in iUIP PCLS but not in normal PCLS. We further found that this perinuclear DNA may activate NF-κB through the cGAS-STING pathway. Although the iUIP lungs, having been exposed to bleomycin once *in vivo*, already showed signs of senescence, we found that further exposure to bleomycin accelerated the cellular senescence.

The critical difference between normal and diseased lungs is oxidative stress due to the generation of ROS and subsequent cell death due to inflammatory conditions. An enlargement of the nucleus is also thought to occur in response to inflammatory tissue damage. From this study, we saw that the acute response to bleomycin may not differ significantly between iUIP and normal lung tissue. However, since iUIP lungs were already in a state of advanced senescence, the further activation of ROS, upregulation of DNA damage repair pathways, and the increase in nuclear permeability likely leads to exacerbated cell death observed in iUIP PCLS. A translocation of cytosolic phospholipase A_2_, cPla_2_ from nucleoplasm membrane to the nuclear envelope may be responsible for the loss of physical tension of the nuclear envelope [30]. This, together with the decreasing lamin B1 expression, may lead to nuclear enlargement and subsequent necrotic cell death, a phenomenon known as cytotoxic edema. Indeed, bleomycin is defined histologically as the causative agent of acute pulmonary edema, but at the cellular level it may be more accurately defined as cytotoxic edema. We hypothesize that bleomycin-induced nuclear swelling leads to leakage of nuclear DNA, which activates the cGAS-STING pathway as a pattern recognition receptor, triggering the observed increase in cell death.

Previous work has shown IPF is a chronic progressive interstitial lung disease marked by senescence due to high levels of P16^INK4A^ [31] and SASP factors such as MMPs [32, 33]. In our iUIP model, increased mRNA expression of senescence markers such as *P16^INK4A^*, various *Mmps*, and *Il6*, can be observed. Therefore, there are common features of cellular senescence in the lungs of patients with IPF and mice with iUIP. Senescence arrests the cell cycle and proceeds without showing apoptosis. In fact, previous data showed no TUNEL-positive cells in iUIP lungs [9]. However, in iUIP PCLS, the generation of ROS by bleomycin administration may alter this senescent state to an unstable state, leading to senescent collapse and cell death such as necrosis. In contrast, in normal PCLS treated with bleomycin, despite the fact that ROS generation was similar to that observed in iUIP PCLS, few TUNEL-positive cells were observed, suggesting resistance to cell death. The cellular ATP levels also decrease in an age-dependent manner and ATP synthesis inhibitors induce necrosis [34]. In fact, iUIP PCLS have half as much extracellular ATP as normal or age-matched control PCLS. Moreover, the decrease in lamin B1 and the subsequent leakage of nuclear DNA into the cytoplasm severely disturbed intracellular maintenance via DNA repair. Thus, an increase in the number of TUNEL-positive cells may cause necrosis in bleomycin-treated iUIP PCLS. Bleomycin treatment is known to induce cytotoxicity followed by apoptosis but has also been shown to induce necrosis at high-dose treatments (10 μg/mL) [5]. However, treatment of the iUIP PCLS with low-dose bleomycin (0.01 μΜ) induced significant LDH released. Therefore, iUIP PCLS are highly sensitive to cytotoxicity, since they produced damage-associated molecular patterns (DAMPs), produced inflammatory markers, and exhibited accelerated senescence, all leading to increased cell death in response to bleomycin treatment. Thus, whereas normal lung cells are able to repair, senescent cells in iUIP PCLS, which are highly sensitive to cytotoxic agents such as bleomycin, may have been unable to repair DNA damage, leading to necrosis rather than apoptosis.

Bleomycin-induced fibrosis has been demonstrated in various *in vitro* studies. Transient *Col1a1* expression in fibroblasts was detected 6 h after bleomycin treatment, which decreased at 24 h [12, 13]. The advantage of our PCLS model is that it recapitulates more closely the microenvironment of the lung, with multiple cell types interacting with one another. In our study, beyond pre-existing fibrosis resulting from the genetic background of iUIP mice, we did not reproduce the transient upregulation of *Col1a1* expression at any time point in PCLS *ex vivo* cultures of normal PCLS without a history of bleomycin treatment. Conversely, when fibrosis cocktails, including TGF-β were added to normal and iUIP PCLS, *Col1a1* expression increased within 5 d [26]. In our study, *Col1a1* expression was not induced by the addition of IL-6 and soluble IL-6 receptor to PCLS. Although persistent inflammation is necessary to induce fibrosis, short-range activation by IL-6, such as in the 5-day culture in our experiments, was not sufficient to induce fibrosis. Another effector of fibrosis, IFN-γ, is a potent inhibitor of *Col1a1* expression by blocking its promoter [12, 35]. We detected increased *Ifng* expression following bleomycin treatment; however, the level of *Ifng* expression was low. Thus, it is difficult to estimate the antifibrotic effects of *Ifng*. Considering all the various factors together, we conclude that bleomycin does not directly induce fibrosis. In this context, secondary effects such as DAMPs from dead cells and/or activation of the NF-κB pathway may induce fibrosis after bleomycin treatment. A detailed analysis of the iUIP model supported this conclusion. Because there was acute cell death but no fibrosis in the 1st week after intratracheal administration of bleomycin, fibrosis with infiltrating lymphoid cells was observed after 2 weeks [9]. These data are supported by the relatively short half-life of bleomycin *in vivo*, which is approximately a few hours to a day. Thus, the direct effects of bleomycin in the body are rather limited and it seems that the fibrotic effects long-term are instead due to the infiltrating inflammatory cells in response to DNA damage, DAMPs, and subsequent necrosis. This is of clinical importance since aging IPF patients often diagnosed with lung cancer, and in this situation anticancer agents such as bleomycin that produce reactive oxygen species and DNA damage pose additional IPF exacerbation risks.

PCLS *ex vivo* culture systems are powerful tools for mimicking *in vivo* conditions. Organoid and tissue-on-a-chip systems have garnered attention as novel *in vitro* drug evaluation platforms that replicate *in vivo* conditions. However, the lungs comprise various cell types, including epithelial cells such as AECI and AECII, club cells, mesenchymal cells, and lymphoid cells. Furthermore, owing to the intricate mesh-like structure and cellular complexity of the lung, accurately reproducing its structural aspects as an organoid remains challenging. Therefore, the PCLS is a valuable tool for assessing drug efficacy and cytotoxicity in lung tissues. On the other hand, an important caveat is that our PCLS experiments use previously frozen tissue, which may lead to some tissue damage once PCLSs are thawed. Previous studies have shown a slight decrease in PCLS viability before and after PCLS freezing [36] as well as decreased mitochondrial activity [37]. In our study, we also found decreased mitochondrial activity in previously frozen samples compared to fresh PCLS; however, no changes in tissue structure or cell population proportions were observed (Supplementary Figure 2). Overall, although using fresh PCLS would be optimal, frozen-thawed PCLSs retain much of the functional and structural features of the lung and remains the most practical and useful model for *ex vivo* culture.

A potential future direction for this work would be to look at the impact of bleomycin treatment in PCLS generated at different stages of lung fibrosis. In our mouse model, fibrosis progresses from the acute phase (NSIP phase), with mild remission (intermediate phase), to the chronic phase (UIP phase), unlike the conventional mouse model [9, 38]. Although PCLS was used in the UIP phase in this study, similar experiments using PCLS in the NSIP and intermediate phases should be conducted in future experiments. In fact, the RNA expression patterns were quite different between the NSIP and UIP phases. For example, *Il-6, Col1a1* and SASP factor expression levels were increased in the NSIP phase but decreased in the UIP phase, whereas collagen deposition was abundant in the UIP phase. Therefore, the effects of bleomycin on PCLS may differ at earlier timepoints. It is also necessary to clarify how anticancer drugs other than bleomycin might affect PCLS, as there remains a need for safer anticancer drugs for IPF patients diagnosed with lung cancer.

## MATERIALS AND METHODS

### Mice and BMS administration in iUIP mice

D1CC×D1BC transgenic mice, bred on a DBA/1J background, were housed in a specific pathogen-free (SPF) animal care facility at Nagoya City University Medical School per institutional guidelines. IP induction using bleomycin and microbubbles was previously described as bleomycin mixed with microbubbles followed by sonoporation (BMS) administration in transgenic mice [9]. Briefly, bleomycin was mixed with an equal amount of microbubbles (Ultrasound Contrast Agent SV-25, NepaGene) and administered through the intratracheal (i.t.) route (40 μL/mouse, 1.28 mg/kg) prior to sonoporation on the chest (1.0 W/cm^2^) for 1 min (Sonitron GTS Sonoporation System, NepaGene). IP induction was monitored by measuring the serum surfactant protein-D (SP-D) levels (R&D Systems, Minneapolis, MN, USA) 2 weeks after induction. The pathological features of UIP are typically emerge 14 weeks after BMS administration, and the iUIP lungs were prepared during this phase. Normal lungs were collected at the same age as when BMS was administered, while age-matched controls were collected at the same age as iUIP lung isolation. D1CC×D1BC transgenic mice without BMS were used as healthy controls. Age-matched control mice were also from the same genetic background without BMS. Therefore, these mice were used for control PCLS.

### Precision-cut lung slices (PCLS) preparation

PCLS from iUIP mice with or without BMS administration was prepared as previously described [26]. Briefly, mouse lungs were inflated with warm 2% low melting agarose (Sigma-Aldrich, Steinheim, Germany) in Hank’s balanced salt solution (HBSS) and solidified for 15 min at 4°C. Each lobe was dissected and embedded in 2% low-melting agarose and cut to a thickness of 300 μm using a vibratome (Compresstome VF-300 OZ; Precisionary, Natick, MA, USA). Slices were cultured in Dulbecco’s modified Eagle medium (DMEM)/F12 (Sigma-Aldrich) with 0.1% fetal bovine serum (FBS), 100 U/mL penicillin, 100 μg/mL streptomycin, and 2.5 μg/mL amphotericin B overnight at 37°C in 5% CO_2_ and frozen with CELLBANKER 1 (Zynogen Pharma, Fukushima, Japan) before use. *Ex vivo* cultures of PCLS samples were treated with 0.01–1 μM bleomycin for 0.5–120 h at 37°C in 5% CO_2_ and replenished at 48 and 96 h. Control slices were supplemented with phosphate-buffered saline (PBS). To determine the effect of the interleukin 6 (IL-6) pathway on fibrosis in *ex vivo* cultures of PCLS, IL-6 (50 ng/mL) and IL-6 receptor (IL6r, 50 ng/mL) were added to PCLS for 24 or 120 h (R&D Systems). The medium was changed every 2 d. Compounds inhibiting the cGAS-STING and Toll-like receptor pathways were tested using 1 μM C-176 (Selleck, Houston, TX, USA) and 1 μM E6446 dihydrochloride (Selleck), respectively.

### Quantitative reverse transcription-polymerase chain reaction (qRT-PCR)

Total RNA was extracted using the ReliaPrep RNA Tissue Miniprep System (Promega, Madison, WI, USA) for PCLS samples and the RNeasy Mini Kit (Qiagen, Venlo, Netherlands) for lung tissues, according to the manufacturer’s instructions. For qRT-PCR, cDNA was synthesized using ReverTra Ace qPCR RT Master Mix with gDNA Remover (TOYOBO, Osaka, Japan). qPCR was performed using specific primers and the Prime Time Gene Expression Master Mix (Integrated DNA Technologies, Coralville, IA, USA). All qPCR probes are listed in Supplementary Table 1. The relative expression of each gene was determined by an internal control using *Hprt* for each sample.

### ROS generation

PCLS samples were cultured with 1 μΜ bleomycin for 30 min at 37°C in 5% CO_2_. ROS generation was assessed using the highly sensitive ROS assay kit dichlorodihydrofluorescein diacetate (DCFH-DA) (Dojin, Kumamoto, Japan), according to the manufacturer’s instructions. The nuclei were stained with Hoechst 33342 (Invitrogen, Carlsbad, CA, USA). Images were captured using a fluorescence microscope (BZ-X710; Keyence, Japan).

### Cellular cytotoxicity

PCLS samples were cultured with 0.01–10 μΜ bleomycin for 4 h for the extracellular ATP assay or for 48 h for the LDH assay at 37°C in 5% CO_2_. Cellular cytotoxicity was assessed using an extracellular ATP assay (Dojin) and LDH assay (Dojin) according to the manufacturer’s instructions. The ATP concentration and percentage of cellular cytotoxicity were determined from the DNA content of each sample using a Quant-iT PicoGreen dsDNA assay kit (Invitrogen).

### Relative cell viability

The PCLS samples were cultured with or without 1 μΜ bleomycin for 48 or 120 h at 37°C in 5% CO_2_. Cell viability was assessed using the CellTiter-Blue Cell Viability Assay (Promega), according to the manufacturer’s instructions. Relative viability was determined by measuring the DNA content of each sample using a Quant-iT PicoGreen dsDNA Assay Kit (Invitrogen).

### Terminal deoxynucleotidyl transferase dUTP nick end labeling (TUNEL) assay

PCLS were cultured with 1 μΜ bleomycin for 48 or 120 h, fixed in 4% paraformaldehyde (PFA) diluted in PBS for 20 min, and embedded in paraffin before 2 μm thick sections were cut. The TUNEL assay of paraffin-embedded PCLS sections was performed using the Mebstain Apoptosis TUNEL kit (MBL, Tokyo, Japan), according to the manufacturer’s instructions. All images were captured using a fluorescence microscope (BZ-X710; Keyence). The percentage of TUNEL-positive cells in the total number of Hoechst-positive cells was calculated by hybrid cell count (Keyence) using ×200 micrograph whole PCLS images.

### Immunohistochemical (IHC) staining

PCLS were cultured with 1 μΜ bleomycin for 48 or 120 h, fixed in 4% PFA diluted in PBS for 20 min, and embedded in paraffin before 2 μm-thick sections were cut. After deparaffinization, the sections were subjected to antigen retrieval by heating in an autoclave in sodium citrate buffer for 5 min and incubation with 0.3% H_2_O_2_ in methanol for 15 min. For IHC, the deparaffinized sections were stained with the following primary antibodies: rat anti-Ki-67 (TEC-3) (DAKO), rabbit anti-LaminB1 and rabbit anti-phospho-histone H2AX (Ser139), rabbit anti-E-cadherin, rabbit anti-vimentin, and rabbit anti-αSMA (Cell Signaling Technology, Danvers, MA, USA), rabbit anti-histone H2A.X, rabbit anti-CD31 (Abcam, Cambridge, UK), rabbit anti-collagen I alpha 1 (Novus biologicals, Centennial, CO, USA), rabbit anti-CD3 (Genemed Biotechnologies, South San Francisco, CA, USA), rat anti-F4/80 (Bio-Rad laboratories, Hercules, CA, USA), rat anti-PTPRC/CD45R (Aviva Systems Biology, San Diego, CA, USA). Histofine Simple Stain Mouse MAX-PO secondary antibodies (Nichirei, Tokyo, Japan) and Opal Multiplex Fluorescent IHC System (Akoya Biosciences, Marlborough, MA, USA) were used according to the manufacturer’s instructions. Nuclei were counterstained with Hoechst 33342 (Invitrogen) and mounted using the ProLong Diamond Antifade Mountant (Invitrogen). All images were captured using a fluorescence microscope (BZ-X710, Keyence) and γH2AX-positive cells and percentage area of collagen type I alpha 1 and αSMA in each PCLS were counted using a hybrid cell count (Keyence).

### Fibrosis ratio

Images showing the area of fibrosis represented in blue (ECM-deposition) by Masson’s trichrome staining (20 minutes for aniline blue staining) were captured by BZ-X analyzer (Keyence) and analyzed by ImageJ, Fiji. Data was calculated as fibrosis ratio, with ECM area divided by total lung tissue area.

### β-galactosidase staining

PCLS were cultured with 1 μM bleomycin for 48 h, fixed in 4% PFA in PBS for 20 min, washed three time in HBSS, and incubated for 1 hour with 20 μM SPiDER-βGal (Dojin) at 37°C. Nuclei were counterstained with Hoechst 33342 (Invitrogen). All images were captured using a fluorescence microscope (Keyence).

### Telomere Q-FISH assay

Telomere length was measured in paraffin-embedded PCLS sections treated with 1 μΜ bleomycin for 48 h using Q-FISH. After deparaffinization, the sections were subjected to antigen retrieval by heating in an autoclave in sodium citrate buffer for 5 min and incubation with 0.3% H_2_O_2_ in methanol for 15 min. PCLS sections were washed before treating with incubated 100 μg/mL of RNase A for 1 h at 37°C followed by incubated 4 μg/mL of protease K for 4 min at 37°C and were washed in tris-buffered saline with Tween (TBS-T) and dehydrated in 70%, 90%, and 100% ethanol. Sections were hybridized with a TelC-Cy3 probe (F1002, HLB Panagene, Daejeon, South Korea) in a hybridization buffer containing 20 mM Na_2_HPO_4_, pH 7.4, 20 mM Tris-HCl, pH 7.4, 70% formamide, 2×SSC, and 0.1 μg/mL fish sperm DNA. Air-dried sections and hybridization buffer were preheated for 5 min at 78°C and 90°C, respectively, then incubated for 10 min at 78°C, and then left overnight at 25°C. After washing in 2×SSC containing 50% formamide, the sections were blocked with 10% goat serum and incubated overnight at 4°C with rabbit anti-phospho-histone H2AX (Ser139) (Cell Signaling Technology) antibody. The sections were washed with TBS-T and incubated with Cy5-conjugated AffiniPure goat anti-rabbit IgG (Jackson ImmunoResearch, West Grove, PA, USA) for 1 h at room temperature. After washing again, the nuclei were counterstained with Hoechst 33342 (Invitrogen) and mounted using the ProLong Diamond Antifade Mountant (Invitrogen). Images were captured using a confocal laser microscope (A1RS+, Nikon).

### Western blotting (WB)

The PCLSs were cultured with 1 μM bleomycin for 48 h, followed by protein extraction. The PCLS samples were vortexed in radioimmunoprecipitation Assay (RIPA) buffer containing 20 mM Tris-HCl (pH 7.4), 150 mM NaCl, 1% Triton, 0.5% deoxycholate sodium, 0.1% sodium dodecyl sulfate (SDS), 1 mM ethylenediaminetetraacetic acid (EDTA), 10 mM 6-O-phosphono-beta-D-galactopyranose (BGP), 10 mM sodium fluoride (NaF), 1 mM sodium orthovanadate (Na3VO4), and a protease inhibitor cocktail. The extracts were sonicated for 15 min and centrifuged at 12,000 rpm for another 15 min. The extract was separated using sodium dodecyl sulfate-polyacrylamide gel electrophoresis (SDS-PAGE) and transferred onto polyvinylidene difluoride (PVDF) membranes. Membranes were blocked with 4% Block Ace (KAC, Kyoto, Japan) at room temperature for 60 min. For WB, the following primary antibodies were used: rabbit anti-lamin B1, rabbit anti-mouse anti-p53, rabbit anti-αSMA (Cell Signaling Technology), and rabbit anti-β-actin and rabbit anti-GAPDH (Proteintech Group, Tokyo, Japan). Enhanced chemiluminescence (ECL)^™^ anti-rabbit horseradish peroxidase (HRP)-linked antibodies were used as secondary antibodies (GE Healthcare, Piscataway, NJ, USA). Each signal was detected using Immunostar LD (Fuji Film, Tokyo, Japan) and an Amersham Imager 600 series (GE Healthcare). Statistical analysis of the expression levels of each protein was performed using an Amersham Imager 600 series (GE Healthcare).

### JC-1 assay

PCLS samples were cultured with 1 μΜ bleomycin for 4 h at 37°C in 5% CO_2_. The JC-1 working solution was stained using a JC-1 MitoMP detection kit (Dojin), according to the manufacturer’s instructions. The nuclei were stained with Hoechst33342 (Invitrogen). Images were captured using a fluorescence microscope (BZ-X710; Keyence). For representative mitochondrial activity and dead cells in fresh or frozen-thawed PCLS samples were used the JC-1 MitoMP detection kit (Dojin) and Dead cell Makeup Deep red -Higher Retention than PI (Dojin).

### Statistics

The results are shown as mean ± standard error (SE). Differences between the vehicle and treatment groups were evaluated using the one-way analysis of variance (ANOVA) followed by Dunnett’s or Student’s *t*-test (Prism10, GraphPad, San Diego, CA, USA). Statistical significance was set at *p* < 0.05.

## Supplementary Materials

Supplementary Figures

Supplementary Table 1
