# Supplementary material for: Bleomycin promotes cellular senescence and activation of the cGAS-STING pathway without direct effect on fibrosis in an idiopathic pulmonary fibrosis model
Source: Aging (Albany NY). 2025 Aug 28;17(8):2189–211. doi: 10.18632/aging.206312 (PMC12422792; doi:10.18632/aging.206312)
Supplement: Supplementary Figures [file aging-17-8-206312-s001.pdf]

SUPPLEMENTARY FIGURES

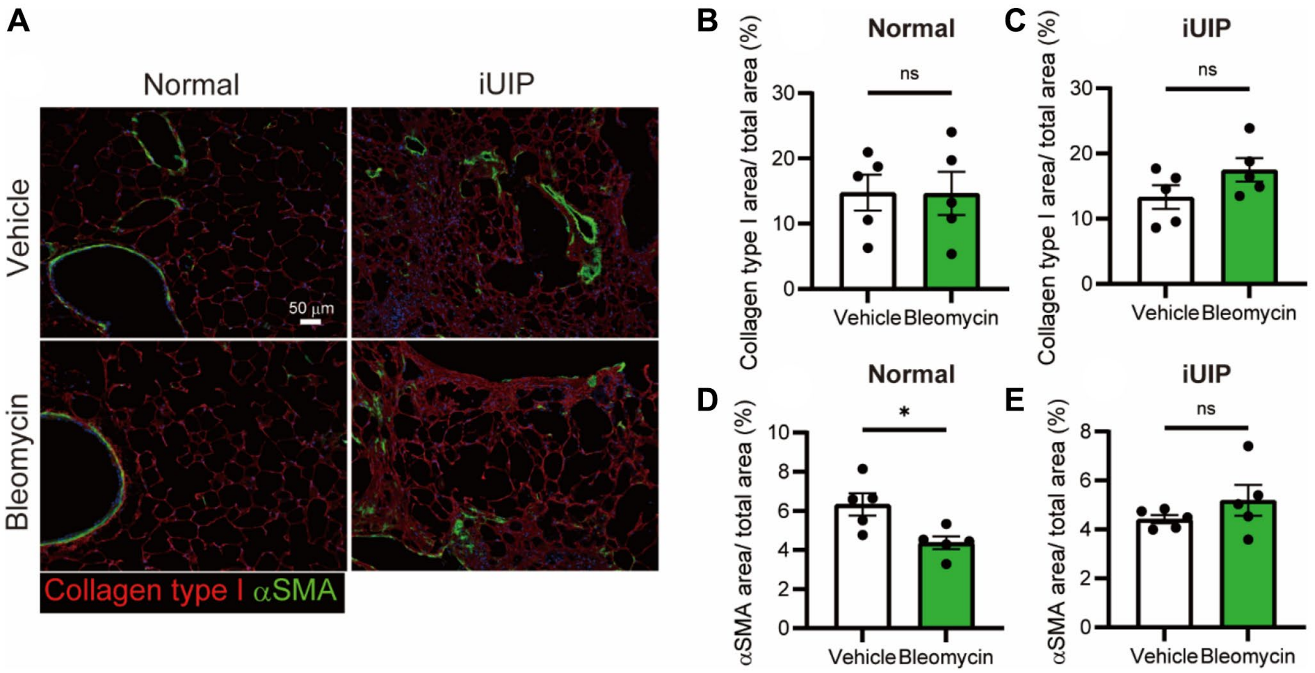

Supplementary Figure 1. (**A**) Immunohistochemical staining of collage type I (red) and a-smooth muscle actin (αSMA; green) in PCLS samples from normal and iUIP treated with the vehicle or 1 μM bleomycin for 48 h. Scale bar indicates 50 μm. (**B**–**E**) Percentage of collagen type I-area and αSMA-area were calculated based on the total area in each PCLS. The results are shown as mean ± SE of five mice at each stage. Asterisks indicate $^{*}P < 0.05$ compared with the vehicle. "ns", not statistically significant.

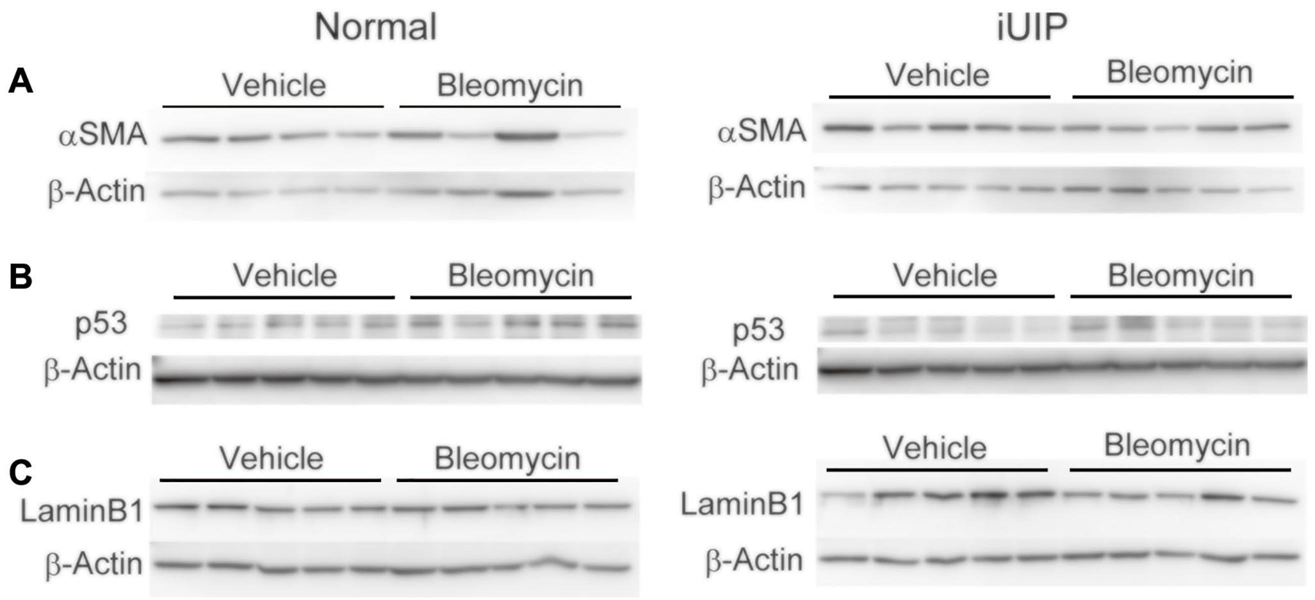

Supplementary Figure 2. PCLS samples were treated with the vehicle or 1 μM bleomycin for 48 h. PCLS samples were lysed and analyzed by WB using antibodies against αSMA (**A**), p53 (**B**), and LaminB1 (**C**). β-Actin was used as an internal control for WB. Four or five mouse PCLS samples at each group.

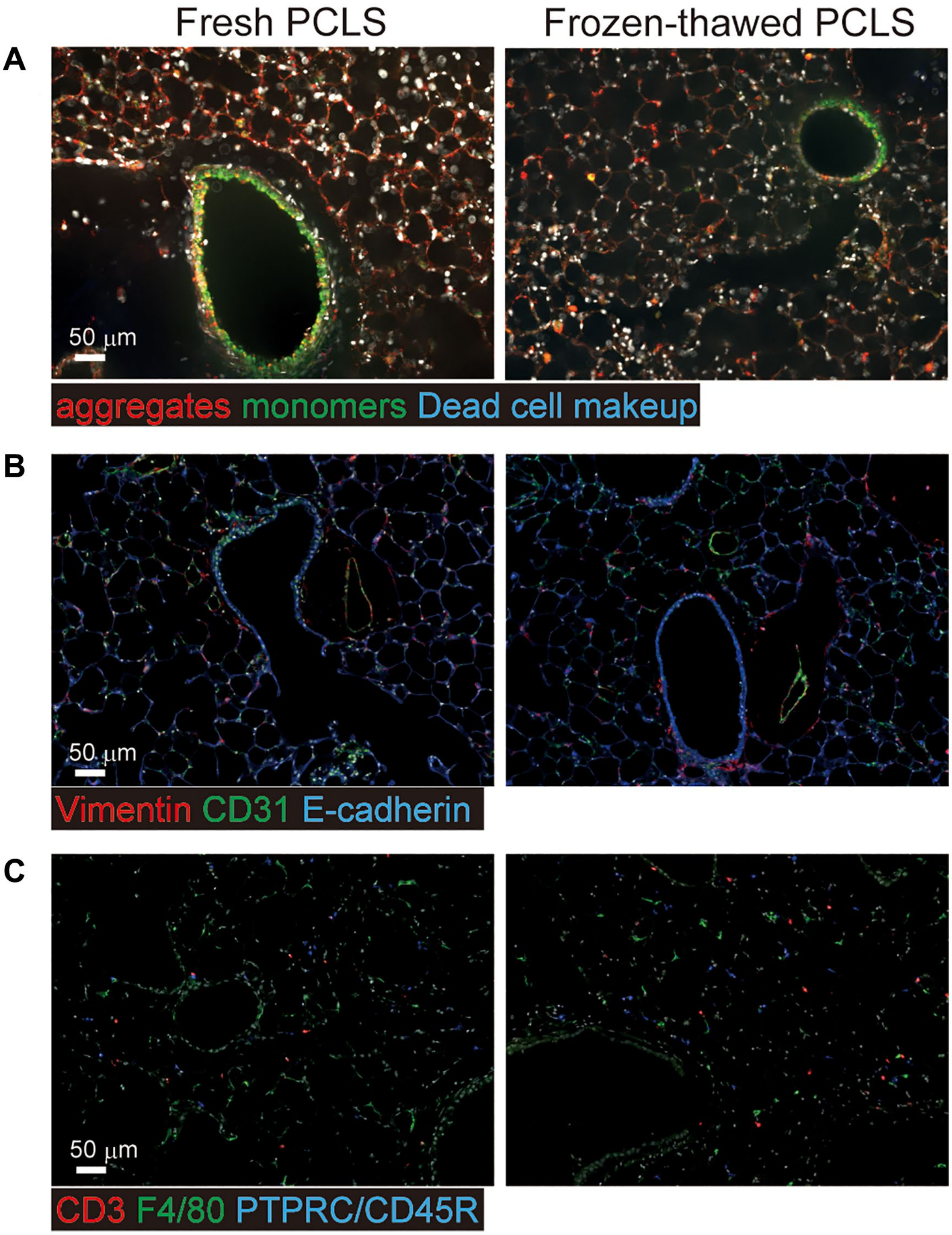

Supplementary Figure 3. Fresh (never frozen) and frozen-thawed PCLS samples were used. (**A**) Representative mitochondrial activity using JC-1 MitoMP (aggregates: red and monomers: green) and cell death using Dead cell makeup (blue) in fresh and frozen-thawed PCLS. (**B**) Immunohistochemical staining of vimentin (red), CD31 (green), and E-cadherin (blue) in fresh or frozen-thawed PCLS samples. (**C**) Immunohistochemical staining of CD3 (red), F4/80 (green), and PTPRC/CD45R (blue) in fresh or frozen-thawed PCLS samples. Scale bars indicate 50 μm.

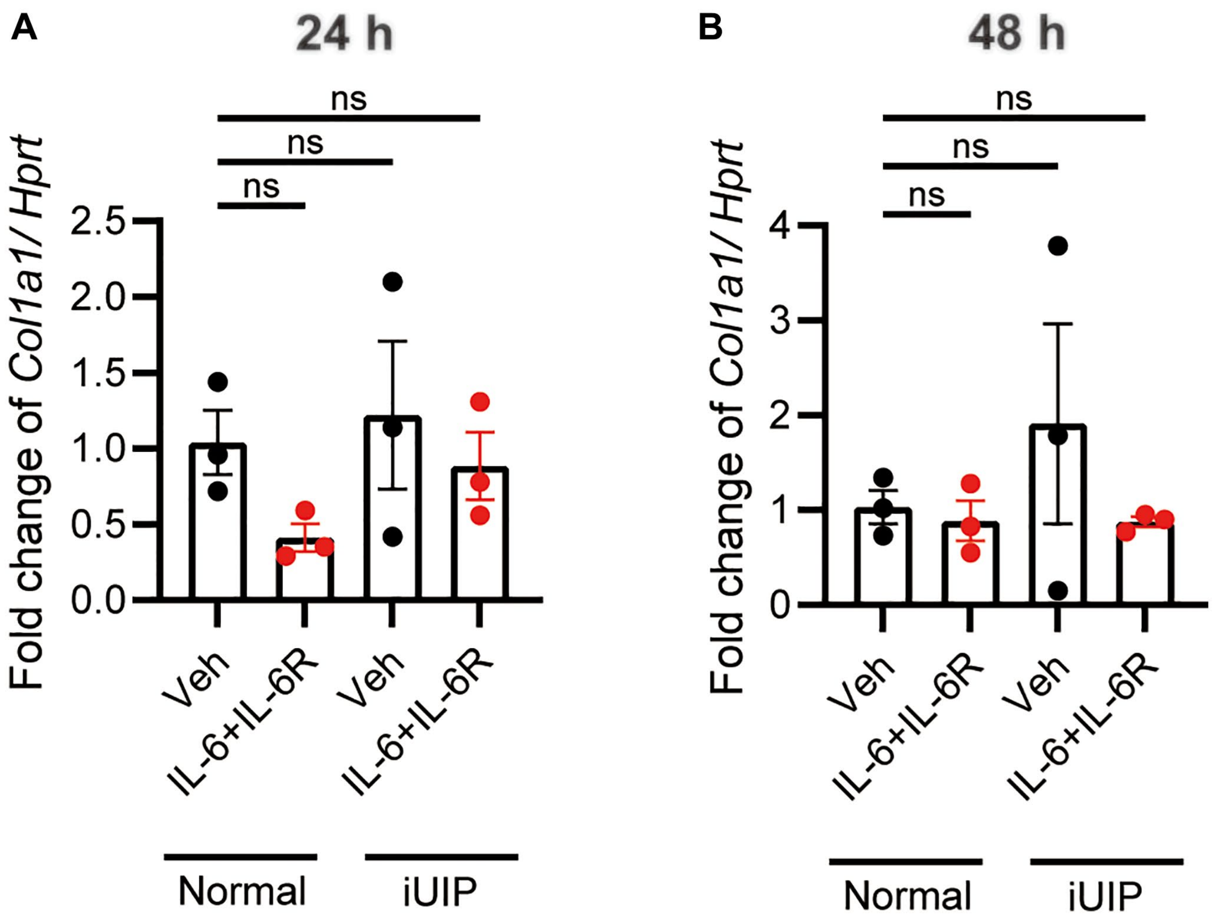

Supplementary Figure 4. (**A**, **B**) PCLS samples were treated with the vehicle or 50 ng/ml IL-6 and 50 ng/ml IL-6R for 24 h (**A**) or 48 h (**B**). *Col1a1* expression was determined using qPCR. *Hprt* was used as an internal control for qPCR. The results are shown as mean ± SE of three (**A**, **B**) mice at each stage. "ns", not statistically significant.

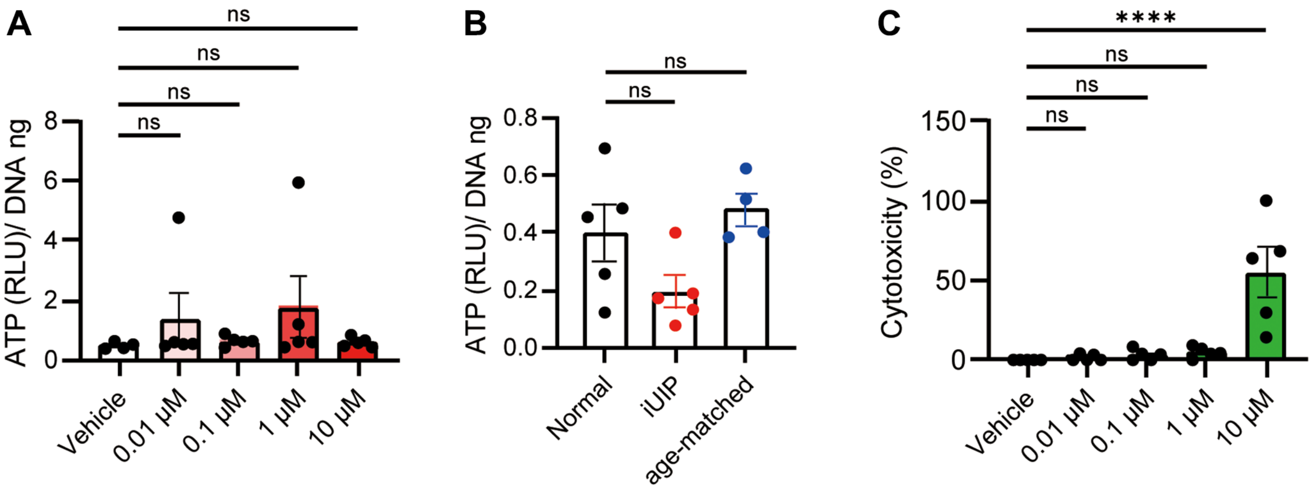

Supplementary Figure 5. (**A**) Extracellular ATP levels were examined in PCLS from age-matched controls treated with the vehicle or 0.01–10 μM bleomycin for 4 h. (**B**) Extracellular ATP was examined in PCLS from normal, iUIP, and age-matched controls treated with the vehicle for 4 h. (**C**) LDH assay was performed in PCLS from age-matched controls treated with the vehicle or 0.01–10 μM bleomycin for 48 h. Values were normalized to DNA content. The results are shown as mean ± SE of five mice at each stage. Asterisks indicate $^{****}P < 0.0001$ compared with the vehicle. "ns", not statistically significant.

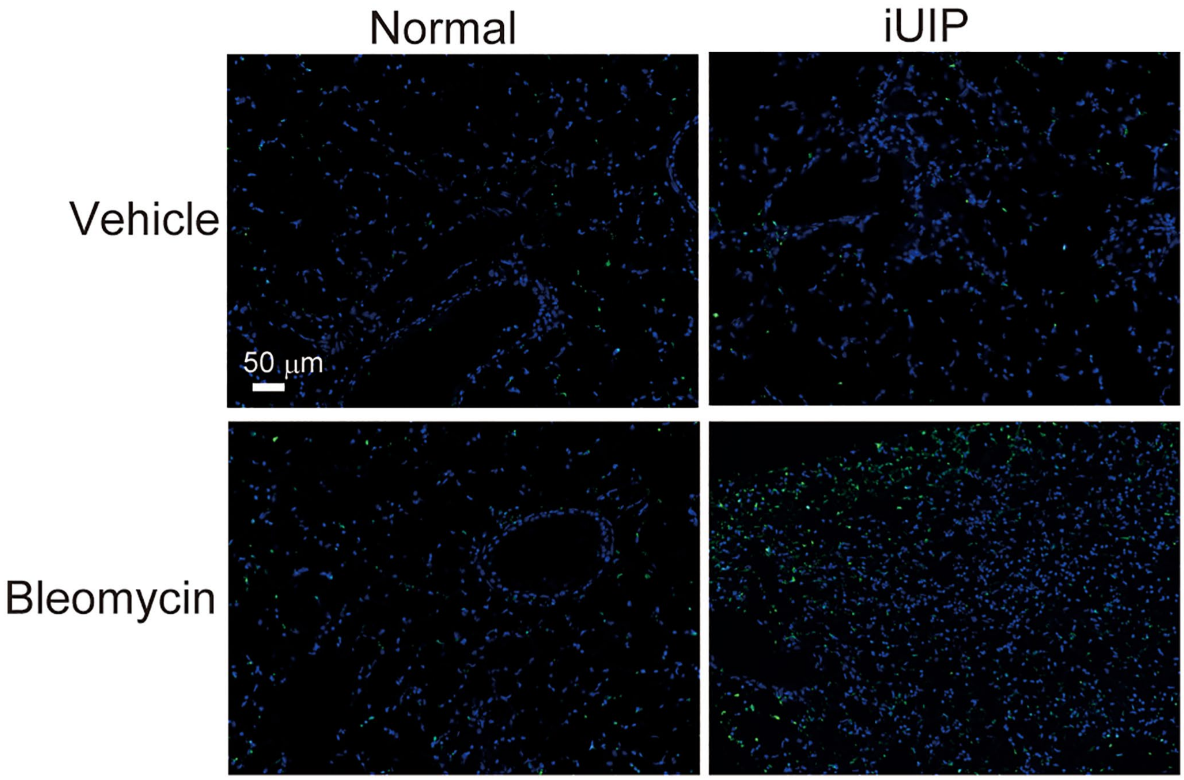

Supplementary Figure 6. Normal and iUIP PCLS were treated with the vehicle or 1 μM bleomycin for 48 h, and paraffin-embedded sections were stained for TUNEL-positive cells. Scale bar indicates 50 μm.

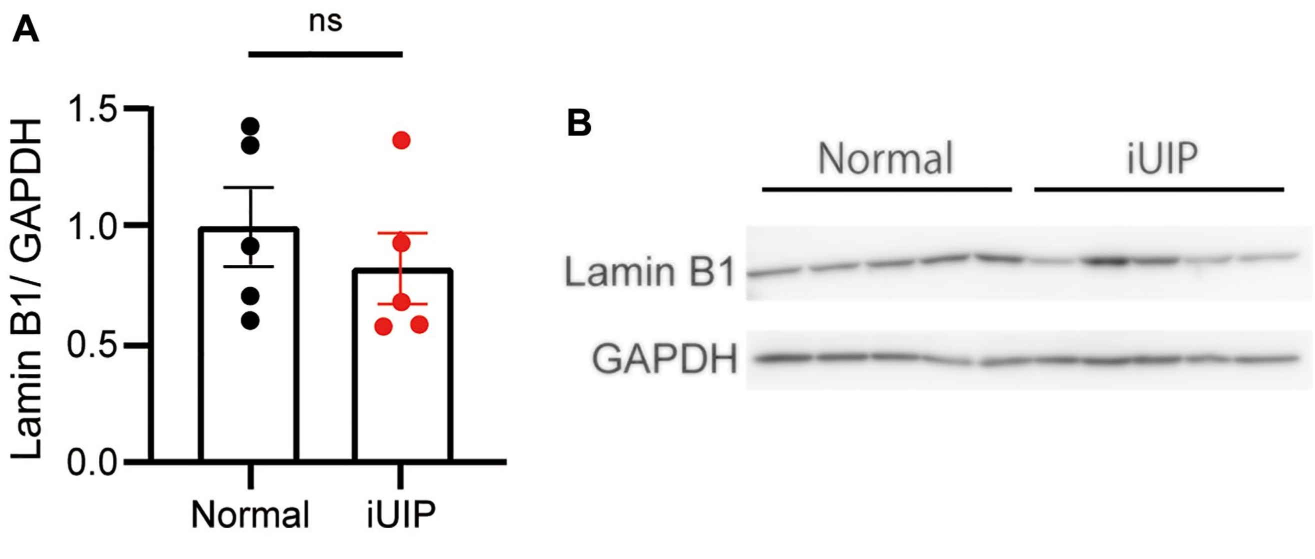

Supplementary Figure 7. (**A**, **B**) Lamin B1 expression in normal and lungs with iUIP was determined using WB. GAPDH was used as an internal control. The results are shown as mean ± SE of five mouse lungs at each group. "ns", not statistically significant.

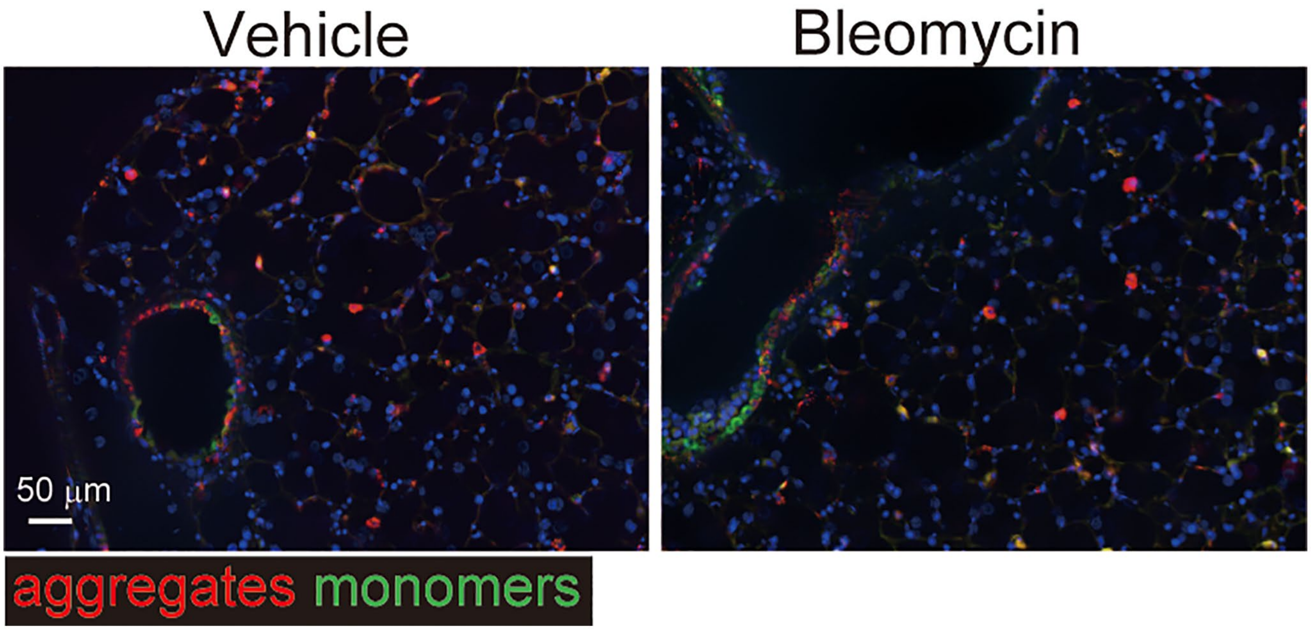

Supplementary Figure 8. Age-matched control PCLS were treated with vehicle or 1 μM of bleomycin for 4 h, and stained using JC-1 MitoMP detection kit. Scale bar indicates 50 μm.
