# Supplementary material for: Bleomycin promotes cellular senescence and activation of the cGAS-STING pathway without direct effect on fibrosis in an idiopathic pulmonary fibrosis model
Source: Aging (Albany NY). 2025 Aug 28;17(8):2189–211. doi: 10.18632/aging.206312 (PMC12422792; doi:10.18632/aging.206312)
Supplement: Supplementary Table 1 [file aging-17-8-206312-s002.pdf]

SUPPLEMENTARY TABLE

Supplementary Table 1. qPCR primers.

Probe	Assay ID	Exon location	Primer sequence
Acta2	Mm. PT. 58. 16320644	7–8	5′-TGCTGACAGAGGCACCACTGAA-3′<br>5′-CAGTTGTACGTCCAGAGGCATAG-3′
Adam17	Mm. PT. 58. 13653280	1–2	5′-CTTTGGTGCCTTTCGTCCT-3′<br>5′-GATGTCGTAGTCTGAGAGCAA-3′
Ccl20	Mm. PT. 58. 13906306	1–2a	5′-CCAGCACTGAGTACATCAACT-3′<br>5′-GTATGTACGAGAGGCAACAGTC-3′
Cxcl5	Mm. PT. 58. 29518961.g	1–2	5′-TTCTGTTGCTGTTCACGCT-3′<br>5′-ATCACCTCCAAATTAGCGATCA-3′
Cxcr1	Mm. PT. 58. 41310733	1–2	5′-TCCCGCACACAAGGAAC-3′<br>5′-TCCCGTGATATTTCCAAATTCTTTC-3′
Col1a1	Mm. PT. 58. 7562513	1–2	5′-CGCAAAGAGTCTACATGTCTAGG-3′<br>5′-CATTGTGTATGCAGCTGACTTC-3′
Hprt	Mm. PT. 39a. 22214828	6–7	5′-CCCCAAAATGGTTAAGGTTGC-3′<br>5′-AACAAAGTCTGGCCTGTATCC-3′
Ifna2	Mm. PT. 58. 45839156.g	1–1	5′-CCTTTCTCTCCTGCCTGAAG-3′<br>5′-CCTTTGATGTGAAGAGGTTCAAG-3′
Ifng	Mm. PT. 58. 41769240	1–2	5′-CTGAGACAATGAACGCTACACA-3′<br>5′-TCCACATCTATGCCACTTGAG-3′
Il6	Mm. PT. 58. 10005566	4–5	5′-AGCCAGAGTCCTTCAGAGA-3′<br>5′-TCCTTAGCCACTCCTTCTGT-3′
Mmp3	Mm. PT. 58. 9719290	4–5	5′-CTCTGGAACCTGAGACATCACC-3′<br>5′-AGGAGTCCTGAGAGATTTGCGC-3′
Mmp7	Mm. PT. 58. 8800692	4–5	5′-GATGCTCACTTTGACAAGGATG-3′<br>5′-GAACAGAAGAGTGACTCAGACC-3′
Mmp8	Mm. PT. 58. 6942600	4–5	5′-GATGCTACTACCACACTCCGTG-3′<br>5′-TAAGCAGCCTGAAGACCGTTGG-3′
Mmp9	Mm. PT. 58. 10100097	8–9	5′-GCTGACTACGATAAGGACGGCA-3′<br>5′-TAGTGGTGCAGGCAGAGTAGGA-3′
Mmp10	Mm. PT. 58. 41830308	10–11	5′-TGCTGCCTATGAGGCTCACAAC-3′<br>5′-GGAGGAAAACCGAGAGTGTGGA-3′
Mmp12	Mm. PT. 58. 31615472	8–9	5′-CACACTTCCCAGGAATCAAGCC-3′<br>5′-TTTGGTGACACGACGGAACAGG-3′
P16^{INK4A}	Mm. PT. 58. 42804808	2–3	5′-CCCAACGCCCCGAACT-3′<br>5′-GCAGAAGAGCTGCTACGTGAA-3′
P19^{ARF}	Mm. PT. 58. 8388138	1–3	5′-TTGAGCAGAAGAGCTGCTACGT-3′<br>5′-GCCGCACCGGAATCCT-3′
S100a8	Mm. PT. 58. 44003402.gs	2–3	5′-ATGACTTCAAGAAAATGGTCACTAC-3′<br>5′-CCACACCCACTTTTATCACCA-3′
Spp1	Mm. PT. 58. 43709208	6–8	5′-AGAATGCTGTGCCTCTGAAG-3′<br>5′-TCGTCATCATCGTCGTCCA-3′

All primers are custom primers provided by Integrated DNA technologies (IDT, Coralville, IA, USA).
